# Reduction of peak plantar pressure in people with diabetes-related peripheral neuropathy: an evaluation of the DH Pressure Relief Shoe™

**DOI:** 10.1186/1757-1146-5-25

**Published:** 2012-10-01

**Authors:** Anita Raspovic, Karl B Landorf, Jana Gazarek, Megan Stark

**Affiliations:** 1 Department of Podiatry and Lower Extremity and Gait Studies Program, La Trobe University, Bundoora, Melbourne, 3086, Australia; 2 Department of Podiatry, The Northern Hospital, 185 Cooper Street, Epping, Melbourne, 3076, Australia; 3 Private Podiatrist, The Melbourne Sports Medicine Centre, Level 4, 250 Collins Street, Melbourne, 3000, Australia

## Abstract

**Background:**

Offloading plantar pressure is a key strategy for the prevention or healing of neuropathic plantar ulcers in diabetes. Non-removable walking casts, such as total contact casts, are currently considered the gold-standard for offloading this type of wound. However, alternative methods for offloading that are more cost effective and easier to use are continually being sought. The aim of this study was to evaluate the capacity of the DH Pressure Relief Shoe™ to offload high pressure areas under the neuropathic foot in diabetes.

**Methods:**

A within-subjects, repeated measures design was used. Sixteen participants with diabetic peripheral neuropathy were recruited and three footwear conditions were evaluated in a randomised order: a canvas shoe (the control), the participants’ own standard shoe, and the DH Pressure Relief Shoe™. The primary outcome was peak plantar pressure, measured using the pedar-X® mobile in-shoe system between the three conditions.

**Results:**

Data analysis was conducted on 14 out of the 16 participants because two participants could not complete data collection. The mean peak pressure values in kPa (±SD) for each condition were: control shoe 315.9 (±140.7), participants’ standard shoe 273.0 (±127.1) and DH Pressure Relief Shoe™ 155.4 (±89.9). There was a statistically significant difference in peak plantar pressure between the DH Pressure Relief Shoe™ compared to both the control shoe (*p* = 0.002) and participants’ standard shoe (*p* = 0.001). The DH Pressure Relief Shoe™ decreased plantar pressures by 51% compared to the control shoe and by 43% compared to participants’ standard shoe. Importantly, for a couple of study participants, the DH Pressure Relief Shoe™ appeared unsuitable for day-to-day wearing.

**Conclusions:**

The DH Pressure Relief Shoe™ reduced plantar pressures more than the other two shoe conditions. The DH Pressure Relief Shoe™ may be a useful alternative to current offloading modalities used in clinical management of diabetic foot ulceration. However, clinical trials are needed to test their effectiveness for ulcer healing and to ensure they are useable and safe for patients in everyday activities.

## Background

Foot ulceration in diabetes has been recognised as a major medical, social and economic problem all over the world [1]. This is not surprising given that approximately 15% of people with diabetes will develop a foot ulcer in their lifetime and that resolution of foot ulcers is often a lengthy and complex process [1]. The burden foot ulceration places on health care systems globally is substantial, with 85% of all diabetes-related amputations being preceded by a foot ulcer [2]. Significant adverse psychological consequences may also result, including diminished psychosocial adjustment to illness, altered satisfaction with personal life, poorer health-related quality of life and depression [3].

Localised tissue trauma, in the presence of clinically-significant sensory neuropathy, is an important risk factor for chronic plantar foot ulceration in people with diabetes [1]. Although, the complex role of biomechanics in diabetes-related foot ulceration is not fully understood, elevated dynamic peak plantar pressure is a strong risk factor for future ulceration, particularly when occurring in conjunction with loss of protective sensation [4]. It stands to reason, therefore, that offloading or reducing elevated peak plantar pressure is important for the successful management of plantar neuropathic foot ulceration in diabetes. A robust body of evidence supports this notion as illustrated by recent high-quality reviews of the literature [5-7].

The salient clinical issue is not whether to use offloading in the management of neuropathic ulceration, but what type/s of offloading modality will achieve the best result in light of the clinical situation and broader patient context. A wide range of offloading modalities are currently available, with studies finding that the ability of these devices to reduce pressure ranges from 20% to 80% when compared to a control [7]. However, a barrier exists to evidence-based offloading practice due to there being limited research and guidelines for offloading modalities, with the exception of total contact casts (TCCs) and other irremovable cast walkers [5]. A study by Wu et al. (2008) showed that the currently accepted gold standard for offloading, the TCC [5,8], was being used regularly by less than 2% of practitioners surveyed, suggesting there are important barriers to the implementation of best-practice offloading [9]. In some instances this may be associated with issues such as lack of training and limited experience with the application of TCCs. In other cases it is likely that there are mitigating circumstances where offloading modalities such as TCCs may not be suitable. Factors such as patient preference, clinical presentation, mobility requirements and social stigma may make use of TCC difficult. In cases where TCCs and other irremovable cast walkers are not appropriate for use, selecting offloading modalities based on evidence is challenging due to the paucity of research.

Alternative methods for off-loading that are effective, economical, easy to use and have little impact on the patient’s lifestyle are continually being sought. The DH Pressure Relief Shoe™ is one such alternative, however to the authors’ knowledge, evidence evaluating its effectiveness at off-loading plantar pressure does not exist. The aim of this study was to evaluate the capacity of the DH Pressure Relief Shoe™ to offload high pressure areas under the neuropathic foot in diabetes.

## Methods

### Participants

This study used a within-subject, repeated measures design. Approval was granted from two Institutional Ethics Committees and informed consent was obtained from all participants prior to their participation in the study. Sixteen participants were recruited to the study, 15 male and 1 female. This sample size was pre-specified and was based on an 80% probability of detecting a clinically meaningful difference between interventions of 100 kPa in peak plantar pressure (standard deviation of 100 kPa and alpha set at 0.05), using an appropriate formula [10]. Participants were recruited via advertisements and direct approach from a local university podiatry clinic and a hospital-based, outpatient high-risk foot service.

Participants were included if they were male or female aged over 18 years and had either a history of, or active plantar neuropathic ulceration. Those perceived at future risk of ulceration were also eligible – risk of ulceration has been defined by Armstrong et al. as a combination of a vibratory perception threshold >25 V and monofilament testing of four imperceptible sites, which has shown to be a highly sensitive and specific method for detecting risk of ulceration [11]. Four participants reported a past history of foot ulceration and one of these participants presented with a current plantar foot ulcer.

Participants were excluded from the study if they were found to have active infection at the wound site, distinguished by the cardinal signs of warmth, redness, pain, tenderness, induration and purulent exudate. Participants were also deemed ineligible if they required the use of a walking aide, had current pain or injury unrelated to the ulceration that affected walking, had an ulcer from other causes, had more than one digit amputated or were unable to speak basic English. Those with acute Charcot’s neuroarthropathy, determined by the clinical signs of unilateral swelling, elevated skin temperature, erythema and joint effusion were also excluded from participating in the study. The characteristics of participants are shown in Table 1.

**Table 1:** Participant characteristics (N = 14)

**Characteristic**	**Value**
Age in years: mean ± SD (range)	65.4 ± 3.3 (48 to 81)
Gender (No.)	13 male : 1 female
Weight in kilograms: mean ± SD (range)	97.2 ± 15.5 (59 to 108)
Diabetes duration in years: mean ± SD (range)	22.7 ± 3.8 (3 to 48)
Previous ulceration	4 yes : 10 no

Note: two cases were excluded due to missing data (footwear issues), therefore the final analysis was conducted on 14 participants.

### Footwear conditions

All participants were measured in the following three footwear conditions:

(i) canvas footwear (Dunlop Volley, Pacific Dunlop Ltd, Melbourne, Australia);

(ii) the participants’ own standard footwear;

(iii) the DH Pressure Relief Shoe™, Royce Medical (now the DH Offloading Post-op Shoe™, Ossur, CA).

**Figure 1:**
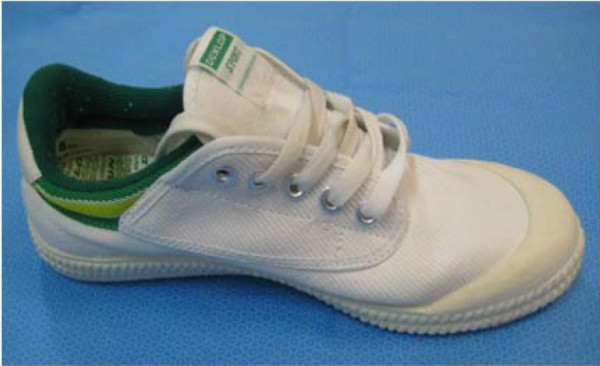
Canvas Dunlop Volley™ (control) shoe.

**Figure 2:**
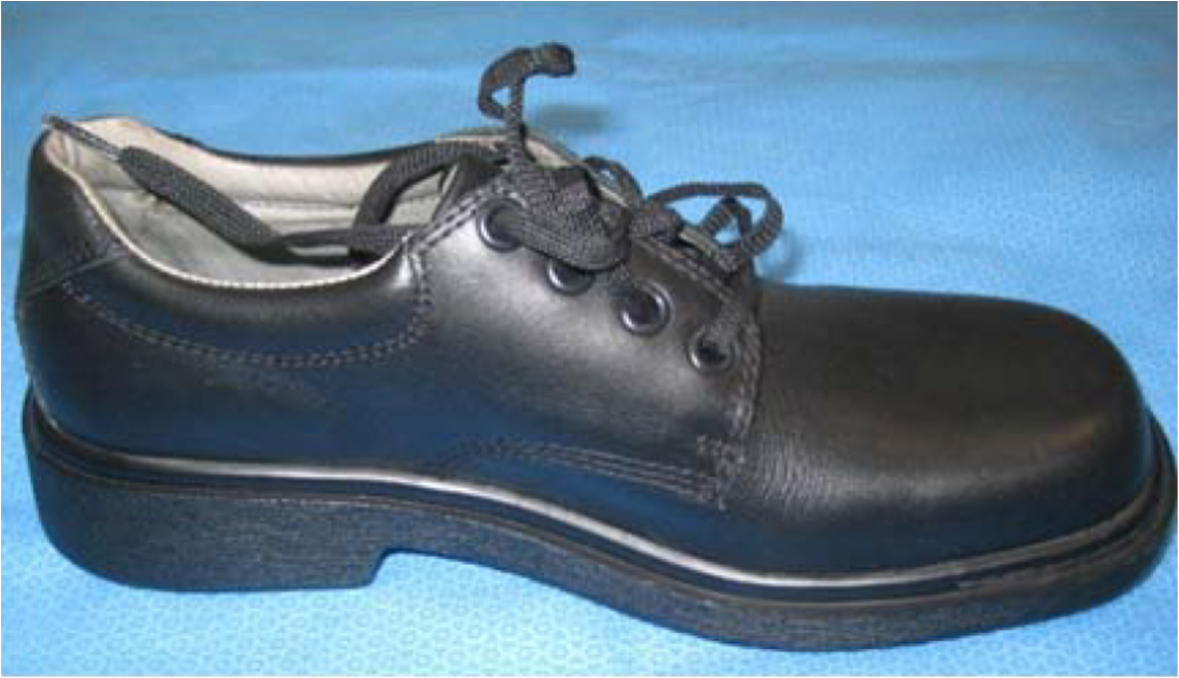
Example of a participant’s standard shoe.

The canvas shoes (Figure 1) were selected as their lightweight construction and flat, thin and flexible soles were considered to have minimal influence on plantar pressures across participants [12]. The participants’ own standard footwear (Figure 2) consisted of the shoes the participant wore most regularly and ranged from standard, extra-depth flat lace-up shoes to customised bespoke footwear. The purpose of this footwear condition was to establish pressure offloading properties of standard footwear to enable a direct comparison to the DH Pressure Relief Shoe™. If the participant wore insoles or orthoses that were additional to any insoles that came with the shoe, these were removed prior to measurement.

**Figure 3:**
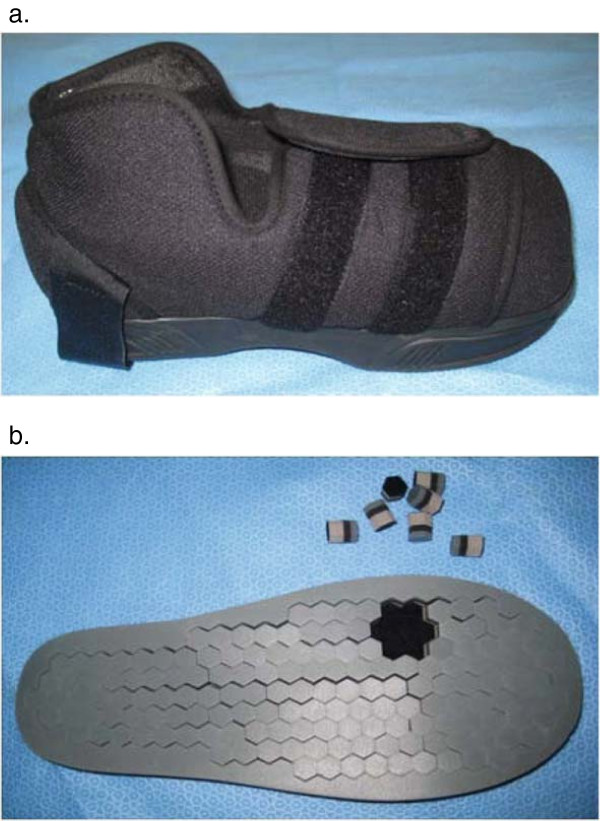
(a) DH Pressure Relief Shoe™ (b) Insole of DH Pressure Relief Shoe™ with hexagonal plugs removed from under the right first metatarsophalangeal joint.

The final shoe condition was the DH Pressure Relief Shoe™ (Figures 3a and 3b), which is a relatively new offloading device designed for people with high pressure or ulceration on the plantar surface of the foot. The DH Pressure Relief Shoe™ is designed for short-term (one to four months) use in the treatment of plantar wounds, such as diabetes-related neuropathic ulceration. The inside of the DH Pressure Relief Shoe™ consists of an insole comprised of hexagonal-shaped plugs, made from three layers that are a combination of mixed density materials including PORON® (Figure 3b). The plugs are 15 mm thick and have a Velcro undersurface to keep them firmly in place but enables their removal over the areas of high pressure as required. The upper of the DH Shoe is a soft synthetic fabric, which is fastened to the foot with large Velcro straps (Figure 3a). For this study, the plugs were removed either under the site of active or previous plantar forefoot or midfoot neuropathic ulceration, or if the participant did not present with a history of neuropathic ulceration, under the right 1st metatarsophalangeal joint, as this is widely accepted as one of the most common sites for neuropathic ulceration [13].

### Randomisation

To minimise potential ordering effects, the three footwear conditions were tested using a random order sequence, generated using Microsoft Excel. The participants and investigators collecting the pressure data could not be blinded to the testing condition that was being assessed in respective trials. However, as the plantar pressure apparatus produces objective data, the investigators thought this would have minimal impact.

### Pressure measuring equipment

Plantar pressures were measured with the in-shoe pedar-X® system (Novel GmbH, Munich, Germany), a reliable, valid and accurate measure of in-shoe pressure [14,15]. The pedar-X® insoles are approximately 2 mm thick and consist of 99 capacitive pressure sensors that are arranged in a grid alignment. Plantar pressure data were sampled at a frequency of 50 Hz. All insoles had been calibrated with the trublu® calibration device prior to the commencement of the study (Novel GmbH, Munich, Germany).

### Measurement protocol

The appropriately sized pedar-X® insole was placed in each shoe condition (i.e. under the foot) for measurement. Prior to the first walking trial of each condition, the pressure insoles were zeroed as described by the manufacturer’s guidelines (Novel GmbH, Munich, Germany). After a familiarisation period of approximately 2 minutes, participants completed three walking trials for each condition. Participants were timed as they walked at a comfortable self-determined speed along a 10 m walkway. If a trial did not fall within 5% of the original walking time, it was eliminated and repeated to minimise the effect of altered walking speed on plantar pressures [16]. To exclude the effect of acceleration and deceleration steps, only the middle steps from each trial of the foot undergoing measurement were included for analysis. An average recording was determined from approximately nine steps (i.e. three steps from three trials) for each condition. In order to meet the independence requirement for statistical analysis, data from only one site on one foot was selected from each participant for analysis [17].

### Outcome measures

The primary outcome measure was peak plantar pressure underneath the selected high pressure site, for each condition. Peak pressure was chosen due to its importance in indicating offloading effectiveness and thus clinical utility. To evaluate if alterations in peak pressure under the mask of interest (i.e. where the DH Pressure Relief Shoe™ plugs were removed) might be attributable to changes in weight bearing area of the plantar surface of the foot, contact area between shoe conditions was analysed as a secondary outcome. We also measured contact time for the whole foot in order to cross check consistency of walking speed.

### Statistical analysis

The plantar pressure data were processed through the Novel-win® program (version 20.3.30). A Novel mask was applied to identify the nominated high pressure site, which formed the primary area for comparison across the three shoe conditions. Data for the variables peak plantar pressure (within mask), contact area (whole foot) and contact time (whole foot) were extracted for analysis using the Statistical Package for the Social Sciences (SPSS) Version 18.0 (SPSS Inc., Chicago, IL). The data were explored for normality of distribution prior to inferential analysis and was within normal limits. One-way repeated measures analysis of variance (ANOVA) with Bonferroni-adjusted post hoc *t*-tests were used to compare measurements between each of the shoe conditions. Comparisons were considered statistically significant if *p* < 0.05.

## Results

### Contact time (whole foot)

To cross check if participants walked at a consistent speed during the trials, differences in whole foot contact time based on the pedar data were evaluated between the three shoe conditions (Table 2). Whilst regularity of walking speed was ensured within ± 5% by the data collection protocol, verification using pedar data was also undertaken due to the important effect of walking speed on peak plantar pressure [16]. No statistically significant difference between contact times (i.e. walking speed) for the whole foot (F_2-12_ = 2.350, *p* = 0.138) were found. Therefore, it can be assumed that any differences in plantar pressures can be attributed to the conditions being analysed.

**Table 2:** Mean, standard deviation and standard error for contact time, peak pressure and contact area for each shoe condition (N = 14)

	**Contact Time (m/s) (whole foot)**		**Peak Pressure (kPa) (mask)**		**Contact Area (cm**^**2**^**) (whole foot)**	
	**Mean**	**SD**	**Mean**	**SD**	**Mean**	**SD**
Canvas	684.5	101.5	315.9	140.7	116.8	20.5
Standard	672.9	88.5	273.0	127.1	124.9	31.7
DH shoe	699.3	96.9	155.4	89.9	127.6	26.7

### Peak pressure differences (mask)

Tables 2 and 3 provide data related to peak pressure changes according to shoe condition. A statistically significant difference in peak plantar pressure was found between shoes (F_2-12_ = 11.813, *p* = 0.001). The DH Pressure-relief Shoe™ reduced peak plantar pressures by 117.7 kPa compared to the standard shoe and 160.5 kPa compared to canvas shoe. This equates to a decrease in peak pressure of 43% and 50% respectively. When compared to the canvas shoe, which was included as the ‘control’ condition, the participant’s own shoes decreased peak pressure by 42.9 kPa or 14% on average, although this did not reach statistical significance.

**Table 3:** Comparison between the shoe conditions for peak pressure and contact area (N = 14)

	**Peak pressure (mask)**			**Contact area (whole foot)**		
	**Mean difference in peak pressure in kPa (95% CI)**	**% difference between shoes**^**#**^	***p*-value**	**Mean difference in contact area in cm**^**2**^**(95% CI)**	**% difference between shoes**^**#**^	***p*-value**
Canvas / Standard	42.9 (−35.6 to 121.3)	14% decrease	0.473	8.1 (−4.9 to 21.1)	7% increase	0.336
Canvas / DH shoe	160.5 (60.1 to 261.0)	51% decrease	0.002	10.8 (2.1 to 19.4)	9% increase	0.014
Standard / DH shoe	117.7 (40.1 to 186.3)	43% decrease	0.001	2.7 (−9.1 to 14.5)	2% increase	1.000

^**#**^ A decrease in the % indicates that the second shoe recorded a lower pressure than the first and an increase in the % indicates that the second shoe recorded a higher pressure than the first.

### Contact area (whole foot)

A statistically significant difference in contact area for the whole foot was found between shoes (F_2-12_ = 5.360, *p* = 0.022). Post hoc tests revealed that there was a significant difference in whole foot contact area between the DH Pressure-relief Shoe™ and canvas shoe (mean difference of 10.8 cm^2^). This increase in contact area equates to the plantar surface of the foot having a 9% larger weight bearing area with the DH Pressure-relief Shoe™ compared to the canvas shoe. There were no differences between the DH Pressure-relief Shoe™ and the standard shoe, or the canvas shoe and standard shoe.

## Discussion

The aim of this study was to evaluate the capacity of the DH Pressure Relief Shoe™ to offload high pressure areas under the neuropathic foot in diabetes. The DH Pressure-relief Shoe™ was selected for investigation in this study as it is currently being marketed and used for offloading plantar foot ulceration in diabetes. To the authors’ knowledge, there has been no independent research that has previously investigated the capacity of the DH Pressure Relief Shoe™ to reduce pressure. In contrast, the DH Pressure Walker™, Royce Medical (now the Active Offloading Walker™, Ossur, CA) a related product in a below-knee walker style, has been investigated previously [18,19]. The DH Pressure Relief Shoe™ is a less bulky version of the DH Pressure Walker™. It is also light, easy to fit and use, and is cost effective at around AUS $100 per unit. Of particular interest is the insole design in the DH Pressure Relief Shoe™, which allows for customisation of offloading by the selective removal of the hexagonal-shaped plugs over high pressure sites. The DH Pressure Relief Shoe™ is designed as a removable intervention and while in some clinical circumstances this might be preferable, research has shown non-removable offloading to be associated with superior healing rates [18-20].

In this study the DH Pressure Relief Shoe™ was shown to reduce mean peak plantar pressure by 117.7 kPa when compared to standard shoes and 160.5 kPa when compared to canvas shoes. This equates to a 43% and 50% reduction respectively, which is comparable in magnitude to studies using specific types of padding and insoles [21,22]. While there is no established pressure cut-off point or threshold above which ulceration will occur, a relationship has been shown to exist whereby the greater the pressure the higher the ulcer risk [11]. With this in mind, we believe that this amount of pressure reduction is clinically important. We powered our study to detect a 100 kPa reduction (or difference between shoes) and the amount of reduction offered by the DH Pressure Relief Shoe™ was well above this. Therefore, the removable plug design insole that is integral to the DH Pressure Relief Shoe™ shows promise for offloading focal plantar pressure. Accordingly, this device may be useful for the treatment of neuropathic ulcers, in the event that these results carry over into trials that use healing as a primary outcome.

It is important to note that the investigators in this project had some concerns about the fixation of the DH shoe in its current form and the stability of some participants while walking. In two cases the ankle straps did not sit flat, which may potentially cause rubbing and irritation to the skin. In another case the medial heel counter was pushed down substantially during gait due to the high degree of rigid foot deformity, rendering the shoe too unstable to be worn during extended periods of walking. Suitability of this device as an offloading modality for some participants was questioned, particularly when dealing with a sub-population known to be at risk of falls. In addition, the aesthetics of the device may be a limitation to its acceptance by some patients. Therefore, the DH Pressure Relief Shoe™ may be a useful alternative to current off-loading modalities but further clinical trials are warranted to determine its safety in everyday activities and relative contraindications.

Several possible explanations exist as to why peak pressures were substantially reduced by the DH Pressure Relief Shoe™. Due to the relatively thick and cushioning make-up of the DH Pressure Relief Shoe™ insoles, it is likely that the plantar surface of the foot sinks into the materials to redistribute force. This, in combination with the removal of the plugs over pressure sites, may explain the levels of offloading recorded. We also found a statistically significant difference in whole foot contact area where the DH Pressure Relief Shoe™ had a 9% greater contact area than the canvas shoe. However, this result did not extend to differences in contact area between the DH Pressure Relief Shoe™ and standard shoe.

Interestingly, peak pressure values in the patients’ own shoes (i.e. the standard shoe condition) did not differ statistically from the canvas control shoe. This finding is consistent with the 2007 evidence-based guidelines of the International Working Group on the Diabetic Foot, which recommend that standard or therapeutic footwear alone should not be used for offloading during ulcer treatment as better modalities are available [7]. It should be noted, however, that the participant’s own shoes were included only to allow comparison of plantar pressures experienced in routinely worn footwear with the DH Shoe. It was not the intention of this study to evaluate footwear as an alternative offloading device.

Our findings should be interpreted in light of the study limitations. Firstly, we measured peak pressure, not healing, as a primary outcome measure and results cannot be directly extrapolated. Pertinent issues that effect healing, such as compliance, functional effectiveness of the device and health-related quality of life, would not be borne out by this study design. Secondly, the study was not designed as a randomised controlled trial, it was an initial exploration of the effects of the DH Pressure Relief Shoe™ on plantar pressure. A larger, high-quality randomised trial with a comparison group is now needed to evaluate the effectiveness of the DH Pressure Relief Shoe™ on ulcer healing. Finally, despite the pedar® system being valid and reliable, it only measures forces acting vertical to the pedar® insole and it is likely that the forces that shoes exert against the plantar surface of the foot are more complex in nature. As the pressure-mapping insoles had to contour the inside of each shoe condition tested, rather than lie flat, they only record resultant force [23,24]. As such, the shear component of such forces are not recorded and some inherent measurement errors are likely to occur [23,24].

## Conclusions

The DH Pressure-relief Shoe™ reduced peak plantar pressure more than the other two shoe conditions tested. Accordingly, it may be beneficial for the treatment or prevention of neuropathic ulcers and may be a useful alternative to current off-loading modalities used for diabetic foot ulceration. Clinical trials are now needed to establish the impact of the device on ulcer healing and prevention, and to ensure they are useable and safe for patients in everyday activities.

## Competing interests

Two authors listed on this publication are members of the Editorial Board of the Journal of Foot and Ankle Research. KBL is currently in the position of Deputy Editor and AR is a member of the Editorial Board. It is journal policy that editors are removed from the peer review and editorial decision making processes for papers they have co-authored.

## Authors’ contributions

AR participated in the inception and design of the study, the data collection, contributed to statistical analysis, and drafted the manuscript. KBL participated in the inception and design of the study, contributed to the statistical analysis, and drafted the manuscript. JG undertook participant recruitment and participated in data collection. MS undertook participant recruitment and participated in data collection. All authors read and approved the final manuscript.
